# Atrial fibrillation burden during in-patient cardiac monitoring after acute ischaemic stroke

**DOI:** 10.1093/esj/aakag079

**Published:** 2026-07-21

**Authors:** Markus G Klammer, Laura Reimann, Oskar Richter, Simone Lieschke, Helena Stengl, Simon Hellwig, Maximilian Schoels, Alexander Nelde, Kersten Villringer, Andreas Meisel, Wolfram Doehner, Christian H Nolte, Christian Meisel, Matthias Endres, Jan F Scheitz

**Affiliations:** Center for Stroke Research Berlin (CSB), Berlin, Germany; Department of Neurology with Experimental Neurology, Charité Universitätsmedizin Berlin, corporate member of Freie Universität Berlin and HumboldtUniversität zu Berlin, Berlin, Germany; Center for Stroke Research Berlin (CSB), Berlin, Germany; Department of Neurology with Experimental Neurology, Charité Universitätsmedizin Berlin, corporate member of Freie Universität Berlin and HumboldtUniversität zu Berlin, Berlin, Germany; Center for Stroke Research Berlin (CSB), Berlin, Germany; Department of Neurology with Experimental Neurology, Charité Universitätsmedizin Berlin, corporate member of Freie Universität Berlin and HumboldtUniversität zu Berlin, Berlin, Germany; Center for Stroke Research Berlin (CSB), Berlin, Germany; Department of Neurology with Experimental Neurology, Charité Universitätsmedizin Berlin, corporate member of Freie Universität Berlin and HumboldtUniversität zu Berlin, Berlin, Germany; Center for Stroke Research Berlin (CSB), Berlin, Germany; Department of Neurology with Experimental Neurology, Charité Universitätsmedizin Berlin, corporate member of Freie Universität Berlin and HumboldtUniversität zu Berlin, Berlin, Germany; Center for Stroke Research Berlin (CSB), Berlin, Germany; Department of Neurology with Experimental Neurology, Charité Universitätsmedizin Berlin, corporate member of Freie Universität Berlin and HumboldtUniversität zu Berlin, Berlin, Germany; Berlin Institute of Health at Charité, Universitätsmedizin Berlin, BIH Biomedical Innovation Academy, BIH Charité Clinician Scientist Program, Berlin, Germany; Center for Stroke Research Berlin (CSB), Berlin, Germany; Department of Neurology with Experimental Neurology, Charité Universitätsmedizin Berlin, corporate member of Freie Universität Berlin and HumboldtUniversität zu Berlin, Berlin, Germany; Berlin Institute of Health, Berlin, Germany; Center for Stroke Research Berlin (CSB), Berlin, Germany; Department of Neurology with Experimental Neurology, Charité Universitätsmedizin Berlin, corporate member of Freie Universität Berlin and HumboldtUniversität zu Berlin, Berlin, Germany; Berlin Institute of Health, Berlin, Germany; Center for Stroke Research Berlin (CSB), Berlin, Germany; Department of Neurology with Experimental Neurology, Charité Universitätsmedizin Berlin, corporate member of Freie Universität Berlin and HumboldtUniversität zu Berlin, Berlin, Germany; Center for Stroke Research Berlin (CSB), Berlin, Germany; Department of Neurology with Experimental Neurology, Charité Universitätsmedizin Berlin, corporate member of Freie Universität Berlin and HumboldtUniversität zu Berlin, Berlin, Germany; Neuroscience Clinical Research Center, Charité Universitätsmedizin Berlin, Berlin, Germany; Center for Stroke Research Berlin (CSB), Berlin, Germany; German Center for Cardiovascular Research (DZHK), partner site Berlin, Berlin, Germany; Berlin Institute of Health Center for Regenerative Therapies, Charité - Universitätsmedizin Berlin, Berlin, Germany; Department of Cardiology, Angiology and Intensive Care Medicine, Deutsches Herzzentrum der Charité, Berlin, Germany; Center for Stroke Research Berlin (CSB), Berlin, Germany; Department of Neurology with Experimental Neurology, Charité Universitätsmedizin Berlin, corporate member of Freie Universität Berlin and HumboldtUniversität zu Berlin, Berlin, Germany; Berlin Institute of Health, Berlin, Germany; German Center for Cardiovascular Research (DZHK), partner site Berlin, Berlin, Germany; Center for Stroke Research Berlin (CSB), Berlin, Germany; Department of Neurology with Experimental Neurology, Charité Universitätsmedizin Berlin, corporate member of Freie Universität Berlin and HumboldtUniversität zu Berlin, Berlin, Germany; Berlin Institute of Health, Berlin, Germany; Center for Stroke Research Berlin (CSB), Berlin, Germany; Department of Neurology with Experimental Neurology, Charité Universitätsmedizin Berlin, corporate member of Freie Universität Berlin and HumboldtUniversität zu Berlin, Berlin, Germany; German Center for Cardiovascular Research (DZHK), partner site Berlin, Berlin, Germany; Deutsches Zentrum für Neurodegenerative Erkrankungen e. V. (DZNE), partner site Berlin, Berlin, Germany; Deutsches Zentrum für psychische Gesundheit (DZPG), Berlin, Germany; Center for Stroke Research Berlin (CSB), Berlin, Germany; Department of Neurology with Experimental Neurology, Charité Universitätsmedizin Berlin, corporate member of Freie Universität Berlin and HumboldtUniversität zu Berlin, Berlin, Germany; Berlin Institute of Health, Berlin, Germany; German Center for Cardiovascular Research (DZHK), partner site Berlin, Berlin, Germany

**Keywords:** atrial fibrillation, atrial fibrillation burden, circadian rhythm, continuous, electrocardiography, ischaemic, stroke, stroke-heart syndrome

## Abstract

**Background:**

Atrial fibrillation (AF) burden, the time spent in AF, is increasingly investigated. Data on its characteristics and temporal patterns during early in-hospital telemetry after ischaemic stroke remain limited.

**Patients and Methods:**

Continuous ECG telemetry was analysed in consecutive patients with MRI-confirmed ischaemic stroke and AF admitted between October 2020 and January 2023 in this observational retrospective cohort trial. Peak AF burden within any 24-h window was categorised as low (≤1 h), medium (>1–6 h), high (> 6 h) or no recorded AF episodes. Associations with clinical and imaging features were assessed using multivariable regression.

**Results:**

Among 392 patients with AF-related stroke (median monitoring duration 68 h, median AF burden 12.0 h), AF burden was low in 30 (7.7%), medium in 68 (17.3%), high in 193 (49.2%) and 101 (25.8%) had no recorded episodes. Left-insular stroke was negatively associated with high AF burden (aOR 0.38, 95% CI, 0.15–0.84, *P* < .05). There was a signal towards a positive association with right-insular stroke (aOR 1.75, 95% CI, 0.90–3.41, *P* = .09). The laterality difference persisted in continuous-scale analyses. First AF episodes clustered in the early evening among low-burden patients, with early morning episodes least common across all groups. Among patients with known stroke onset (*n* = 102), 49 exhibited AF confined to the first 48 h.

**Conclusion:**

AF burden varies widely during early in-hospital monitoring after ischaemic stroke and seems to differ according to insular lesion laterality. Our findings are hypothesis-generating and highlight the need for integrating lesion location, circadian and arrhythmia dynamics to characterise AF burden after stroke.

## Graphical Abstract

**Graphical Abstract:**
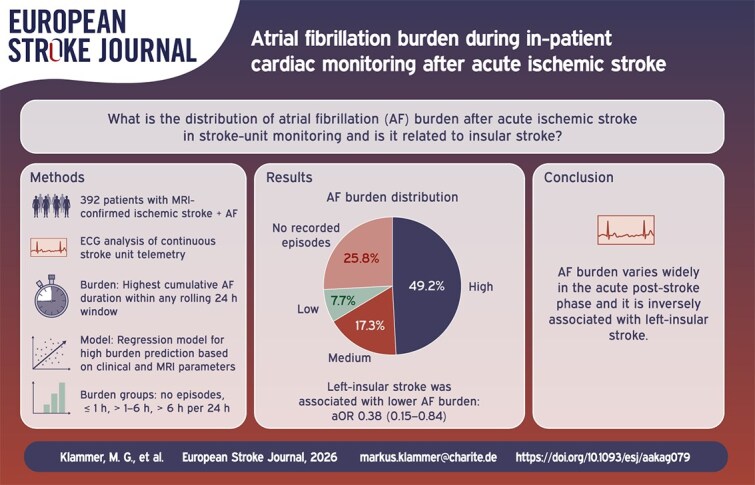

## Introduction

Atrial fibrillation (AF) is a common stroke risk factor.^1^^,^^2^ Traditionally regarded as binary (present/absent), AF is increasingly recognised as a spectrum, with burden varying substantially between individuals.^3^ AF burden, the time spent in AF over a defined period, is positively associated with embolic risk.^4^ Major trials have assessed AF burden mostly in non-stroke patients with pacemakers, implantable loop recorders (ILR) or Holter monitoring (ASSERT,^5^ TRENDS^6^). Individual studies in stroke patients exist,^7^^,^^8^ but burden definitions vary, and data on AF burden during in-hospital stay after acute stroke remain particularly scarce. This gap is relevant because stroke, especially insular stroke, can trigger cardiac complications, including AF and myocardial injury, via autonomic disruption^9^ and insular laterality differentially affects cardiac outcomes.^10–12^ An individualised approach to embolic risk, for example, by assessing AF burden, may be warranted.^13^

Studying the AF burden and episode dynamics in the earliest phase after ischaemic stroke is challenging. Stroke unit data have historically been difficult to analyse due to storage constraints and competing monitoring needs, but advances in data warehousing now enable large-scale stroke unit monitoring analysis.^14^ Unlike ILRs or Holter monitors, which are feasible in only a minority of stroke patients, stroke unit monitoring covers a broader population and may help identify AF subtypes or stroke-induced arrhythmias, informing risk stratification and treatment.

We aimed to characterise short-term AF burden and episode dynamics in ischaemic stroke patients with AF (known AF or AF detected after stroke [AFDAS]) using continuous ECG monitoring on a stroke unit. Specifically, we sought to (1) quantify AF burden during stroke-unit monitoring, (2) examine temporal and circadian patterns of AF episodes, (3) analyse heart rate (HR) dynamics during AF and (4) explore associations between AF burden and stroke characteristics, particularly insular involvement.

## Methods

This retrospective, observational cohort study used routinely collected patient data and data from the CORONA-IS trial (Cardiomyocyte Injury After Acute Ischemic Stroke).^15^ All existing parameters were available to the investigators. Consecutive patients with MRI-confirmed ischaemic stroke and available cardiac monitoring, admitted to a comprehensive stroke unit at a university hospital in Berlin, Germany, between October 2020 and January 2023, were included. Exclusion criteria were sinus rhythm, intracranial bleeding, non-stroke diagnoses, missing AF status or low MRI quality. Ethics approval was granted by the Charité Universitätsmedizin Berlin Ethics Committee (EA4/239/23).

ECG data were extracted from stroke unit monitors and stored in the Data Warehouse Connect (DWC, Philips). Only pre-identified patients were extracted. Use of the DWC was approved by the Ethics Committee (EA1/376/20).

AF burden was calculated as the sum of AF episode durations within a rolling 24-h window applied hourly. Maximum 24-h AF burden was classified as low (≤1 h), medium (>1–6 h) or high (>6 h, classification adapted from Sposato et al. suggested for long-term ECG monitoring^13^). Patients with documented AF but no recorded episodes were analysed separately (“no recorded episodes”). The rolling window approach ensured comparability across variable monitoring durations. Monitoring time was defined from the first to last recorded cardiac activity, including gaps.

To identify predictors of high AF burden (>6 h/24 h), univariate logistic regression analyses were performed for all demographic, clinical and imaging variables. Variables with *P* < .05 were entered into a multivariable logistic regression model, additionally adjusted for age, sex and lesion volume. Stroke affecting the insular cortex was entered as left, right and not affected (reference). To assess insular laterality effects, sensitivity analyses were conducted with right- and left-sided insular stroke entered separately (thereby modelling, eg, left insular stroke against non-insular and right insular stroke as reference) and adjusted for ipsilateral middle cerebral artery (MCA) infarction.

Model robustness was tested using AF burden as a continuous log-transformed outcome in linear regression, applying the same two-step modelling approach. Regression coefficients (β) with 95% CI are presented in the Supplementary material.

Further methodological details are provided in the Supplementary Methods.

Summary statistics are presented as mean ± SD, median (IQR) or percentages. Parametric or nonparametric tests compared continuous variables; Chi-squared or Fisher’s exact test compared categorical variables. Analyses used R 4.4.1 with ggplot2.

## Results

**Figure 1:**
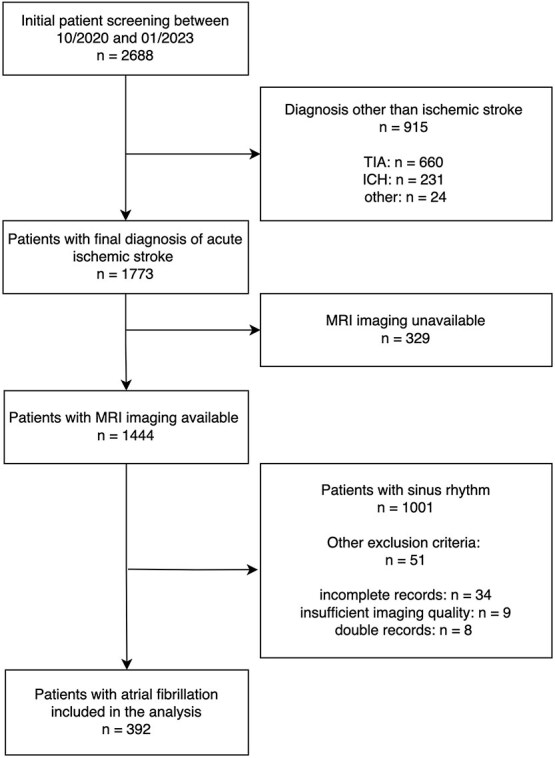
Flow chart of cohort selection.

Initial screening identified 2688 patients admitted to the stroke unit between October 2020 and January 2023 (Figure 1). After excluding non-ischaemic strokes (*n* = 915), patients in sinus rhythm (*n* = 1,001), those without MR imaging (*n* = 329), insufficient imaging quality (*n* = 9) and incomplete/double records (*n* = 42), 392 ischaemic stroke patients with AF remained. Median age was 81 years (IQR 77–86), median NIHSS 4 (2–9) and 53.1% were female.

**Figure 2:**
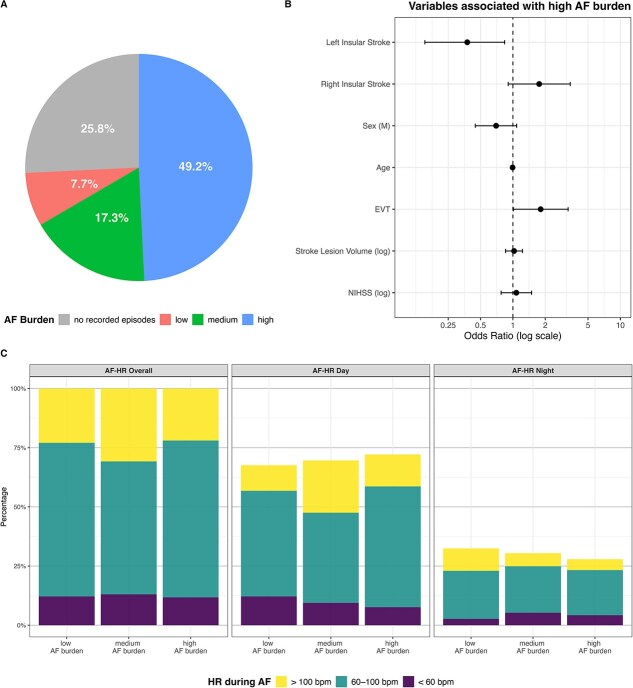
AF burden, high-burden features and circadian HR patterns. (A) Proportion of patients with low (<1 h/24 h), medium (1–6 h/24 h) and high (>6 h/24 h) AF burden. (B) Variables associated with high AF burden. Left-sided insular stroke is independently inversely associated. (C) Median HR during AF episodes by burden: overall, diurnal (6–22 h) and nocturnal (22–6 h). Medium-burden patients have a higher proportion of tachycardic episodes than low- or high-burden patients.

Table 1 shows patient characteristics by AF burden. Median cardiac monitoring was 64.6 hours (IQR 41.9–99.8). AF burden ranged the full span from 0 to 24 h (median 12 h, IQR 3.7–20.0) and was high in 193 patients (49.2%), medium in 68 (17.3%), low in 30 (7.7%) and 101 (25.8%) had no recorded episodes (Figure 2A). High burden patients had higher admission NIHSS (5 vs. 2 and 4, *P* < .01), larger stroke volume (3.2 vs. 1.0 and 2.9 mL, *P* < .05) and higher EVT rate (31.6% vs. 16.7% and 26.5%, *P* < .05). Right insular stroke was most frequent in this group (18.1% vs. 6.7% and 10.3%, *P* = .09). A comparison of patients with high burden versus those without high burden is displayed in Table S1.

**Table 1:** Baseline characteristics of AF patients.

	AF burden			
Variable	No episodes	Low < 1 h	Medium 1–6 h	High > 6 h
**N**	101 (25.8)	30 (7.7)	68 (17.3)	193 (49.2)
**Age (year)**	79 (76–84)	83.5 (77–86)	84 (80–88)	82 (76–86)
**Female**	44 (43.6)	15 (50.0)	43 (63.2)	108 (56)
**Length of monitoring (h)**	64.6 (41.9–99.8)	48 (37.1–80)	73.3 (41.1–135.4)	70.5 (44.1–97.3)
**NIHSS on admission**	3 (1–7)	2 (1–5)	4 (2–9)	5 (2–11)
**IVT**	11 (10.9)	5 (16.7)	18 (26.5)	38 (19.7)
**EVT**	17 (16.8)	3 (10.0)	17 (25.0)	61 (31.6)
**Cardiovascular risk factors**				
**Arterial hypertension**	83 (82.2)	25 (83.3)	50 (73.5)	155 (80.3)
**Diabetes mellitus**	28 (27.7)	6 (20.0)	13 (19.1)	50 (25.9)
**Dyslipidaemia**	75 (74.3)	20 (66.7)	46 (67.6)	129 (66.8)
**Coronary artery disease**	26 (25.7)	3 (10.0)	17 (25.0)	28 (14.5)
**History of myocardial infarction**	10 (9.9)	3 (10.0)	4 (5.9)	14 (7.3)
**Ipsilateral ICA stenosis > 50%**	11 (10.9)	1 (3.3)	3 (4.4)	6 (3.1)
**CHA2DS2-VA score**	3 (3–4)	3 (3–4)	3 (3–4)	3 (2–4)
**Known AF**	83 (82.2)	25 (83.3)	49 (72.1)	155 (80.3)
**AFDAS**	18 (17.8)	5 (16.7)	19 (27.9)	38 (19.7)
**Active intake of OAC**	56 (55.4)	10 (33.3)	28 (41.2)	96 (49.7)
**Laboratory values**				
**Admission hs-cTnT (ng/L)**	21.5 (14–44)	25 (16–36.8)	26 (17–45.5)	23 (15–35)
**CRP (mg/L)**	2.8 (1.1–10.2)	4 (1.5–8.2)	3.8 (2–14.3)	3.7 (1.7–14.1)
**Leukocytes (10^9^/L)**	8.3 (6.6–10.2)	9.4 (6.7–11.4)	9.1 (7.8–11.7)	8.7 (7–10.9)
**GFR (mL/min/1.73 m^2^)**	60 (46.8–79.2)	54 (44–66)	60 (51–77.5)	58.5 (43.8–74.2)
**HR pattern**				
**SVES in 24 h**	302.8 (39.7–1021.9)	1259.5 (301.5–2360.8)	458.2 (159.4–1605)	910.3 (337–1620.8)
**HR overall**	71.8 (65.8–82.0)	73.3 (63–82.5)	78.8 (69.6–88.5)	78.2 (69.6–89.9)
**Stroke characteristics on MRI**				
**Right anterior circulation stroke**	19 (18.8)	4 (13.3)	22 (32.4)	53 (27.5)
**Left anterior circulation stroke**	29 (28.7)	5 (16.7)	14 (20.6)	43 (22.3)
**Posterior circulation stroke**	18 (17.8)	6 (20.0)	8 (11.8)	38 (19.7)
**Stroke in multiple vascular territories**	35 (34.7)	15 (50.0)	23 (33.8)	59 (30.6)
**Haemorrhagic transformation**	18 (17.8)	4 (13.3)	20 (29.4)	50 (25.9)
**Stroke lesion volume (mL)**	1.7 (0.3–8.8)	1 (0.2–3.8)	2.9 (0.4–11.5)	3.2 (0.4–16.8)
**Highest quartile of lesion volume**	29 (28.7)	7 (23.3)	23 (33.8)	75 (38.9)
**Right insular stroke**	10 (9.9)	2 (6.7)	7 (10.3)	35 (18.1)
**Left insular stroke**	13 (12.9)	4 (13.3)	10 (14.7)	18 (9.3)

Abbreviations: OAC = oral anticoagulants; AF = atrial fibrillation; AFDAS = atrial fibrillation detected after stroke; CRP = C-reactive protein; EVT = endovascular treatment; GFR = glomerular filtration rate; HR = heart rate; hs-cTnT = high-sensitivity cardiac troponin T; ICA = internal carotid artery; IVT = intravenous thrombolysis; MRI = magnetic resonance imaging; NIHSS: National Institutes of Health Stroke Scale; SVES = supraventricular extrasystoles. Summary statistics are displayed as median (IQR) or *n* (%).

Regression modelling tested variables associated with AF burden. To ease clinical interpretability and improve statistical power, we dichotomised the outcome (high AF burden >6/24 h vs. < 6/24 h). In univariate logistic regression, high AF burden was associated with higher log NIHSS (OR 1.30, 95% CI, 1.02–1.66, *P* < .05), EVT (OR 2.07, 95% CI, 1.30–3.29, *P* < .01) and right insular stroke (OR 2.47, 95% CI, 1.37–4.51, *P* < .01), and we observed a signal towards an inverse association with left insular cortex stroke (OR 0.48, 95% CI, 0.20–1.03, *P* = .08). Multivariable models confirmed EVT as positively associated (aOR 1.81, 95% CI, 1.01–3.27, *P* < .05) and left insular stroke as negatively associated with high AF burden (aOR 0.38, 95% CI, 0.15–0.84, reference: non-insular stroke, *P* < .05, Figure 2B). There was a signal towards a positive association with right insular cortex stroke (aOR 1.75, 95% CI, 0.90–3.41, reference: non-insular stroke). Modelling insular stroke as dichotomised variables, right-sided insular stroke (reference: all other strokes incl. Left-sided insular stroke) was positively associated (aOR 5.50, 95% CI, 2.02–16.42, *P* < .01) and left-sided insular stroke (reference: all other strokes incl. Right-sided insular stroke) was negatively associated (aOR 0.19, 95% CI, 0.07–0.51, *P* < .01; see Figure 2B and Supplementary material) with high AF burden.

Continuous-scale regression analyses yielded consistent results (Supplementary Results).

Among patients with recorded episodes, high AF burden was associated with more and longer episodes (median 8–107 min, *P* < .001, Table 2). Heart rate characteristics by burden group are shown in Figure 2C and Supplementary Results. Time from stroke onset to first AF episode was shortest in high-burden patients (12.4 vs. 22.7 h, *P* < .05, Table 2).

**Table 2:** Summary of AF characteristics and cardiac monitoring parameters.

Variable	All	AF burden			*P* _ overall_
		Low < 1 h	Medium 1–6 h	High > 6 h	
**Patient count**	291 (100)	30 (10.31)	68 (23.37)	193 (66.32)	
**Time spent in AF over 24 h (h)**	4.2 (1.4–10.2)	0.1 (0.1–0.2)	1.2 (0.6–1.9)	7.9 (4.2–14.5)	<.001
**% of monitoring time spent in AF**	17.5 (5.8–42.4)	0.5 (0.3–1)	5 (2.5–7.8)	33 (17.3–60.4)	<.001
**Number of episodes**	10 (4–18)	2 (1–3)	5 (2–13)	13 (8–23)	<.001
**Median length of AF episode (min)**	73.7 (30.9–137.8)	8.1 (6.8–17.9)	29.8 (13–59.6)	107.2 (60.1–160.9)	<.001
**Shortest AF episode (min)**	5.7 (3–7.8)	5.4 (3.9–5.8)	5.8 (4–7.2)	5.8 (2.6–8)	.51
**Longest AF episode (min)**	272.9 (111.3–449.3)	13.3 (8.8–29.8)	80.3 (45.6–131.9)	359.9 (236.3–542.2)	<.001
**Overall HR during AF episodes**	84.9 (71.6–102)	83.1 (63–103.9)	92.5 (75.1–121.6)	82.1 (71.7–98.4)	<.05
**HR during daytime AF episodes**	87.2 (72.5–103.4)	84.7 (62.9–102.6)	93.7 (75.7–116.7)	85.5 (72.5–100.6)	.08
**HR during nighttime AF episodes**	86.1 (71.4–101.9)	83.2 (63.2–104.2)	92.0 (74.6–117.7)	84.6 (71.4–99.8)	.11
**Overall HR during SR**	75.0 (66.6–87.4)	70.8 (62.1–82.2)	74.1 (68.1–85.9)	76.2 (67.3–88.1)	.22
**HR during daytime SR**	76.6 (68.2–88.8)	72.1 (65.9–83.5)	75.5 (68.9–89.2)	78.0 (68.5–89.6)	.3
**HR during nighttime SR**	76.8 (67.1–88.2)	72.9 (63.2–82.1)	76.2 (68.8–89.9)	77.3 (67.4–88.3)	.23
**% of AF episodes with normal HR**	59.7 (40.5–72)	52.7 (31.7–69.8)	51.5 (24.2–71.7)	63.5 (46–72.1)	<.01
**% of AF episodes with bradycardia**	5.6 (1–22.8)	7.9 (0.5–29.5)	2.6 (0.5–17.5)	7.4 (1.5–23.0)	.09
**% of AF episodes with tachycardia**	22.8 (3.3–53.8)	21.9 (4.4–59.2)	34.3 (6.3–73.5)	20.8 (2.5–47.7)	.06
**Time to first AF episode after stroke onset (h)**	14.3 (5.6–36.1)	22.7 (8.9–45.3)	16.5 (8–51.6)	12.4 (4.8–27.1)	<.05
**Time to cardiac monitoring after stroke onset (h)**	9.3 (3.8–22.5)	13.5 (4.2–41.9)	12.8 (4.7–26.9)	7.7 (3.7–18.7)	.07

Abbreviations: AF = atrial fibrillation; AFDAS = AF detected after stroke; HR: heart rate; SR = sinus rhythm. AF episode characteristics during in-patient cardiac monitoring (101 patients without any recorded episodes were excluded from this table).

**Figure 3:**
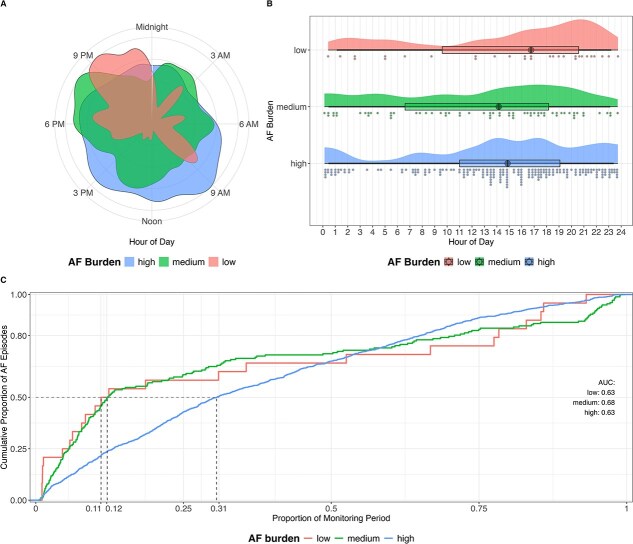
AF episode distribution during stroke unit monitoring stratified by AF burden (low: < 1 h/24 h, medium: 1–6 h/24 h, high: > 6 h/24 h). (A) Circadian distribution. Low-burden episodes cluster 21–24 h; medium- and high-burden episodes are more evenly distributed. (B) Timing of first episodes. Low-burden AF tends to occur late afternoon/early evening; early morning shows the lowest frequency across all groups. (C) Cumulative distribution over normalised monitoring. In low- and medium-burden patients, 50% of episodes occurred within the first 11% and 12% of monitoring; high-burden patients reached 50% after 31% or normalised monitoring period. AUC > 0.5 for all groups indicates left-shifted episode timing.

Circadian distribution of AF episodes showed low-burden patients peaking 9 pm–midnight, whereas high-burden patients had a more even distribution (Figure 3A). First episodes clustered late afternoon/early evening in low-burden patients, and early morning was least likely across all groups (Figure 3B). Cumulative episode distribution showed early occurrence in low/medium-burden patients (50% within first 11%–12% of monitoring), whereas high-burden episodes distributed more uniformly (Figure 3C).

**Figure 4:**
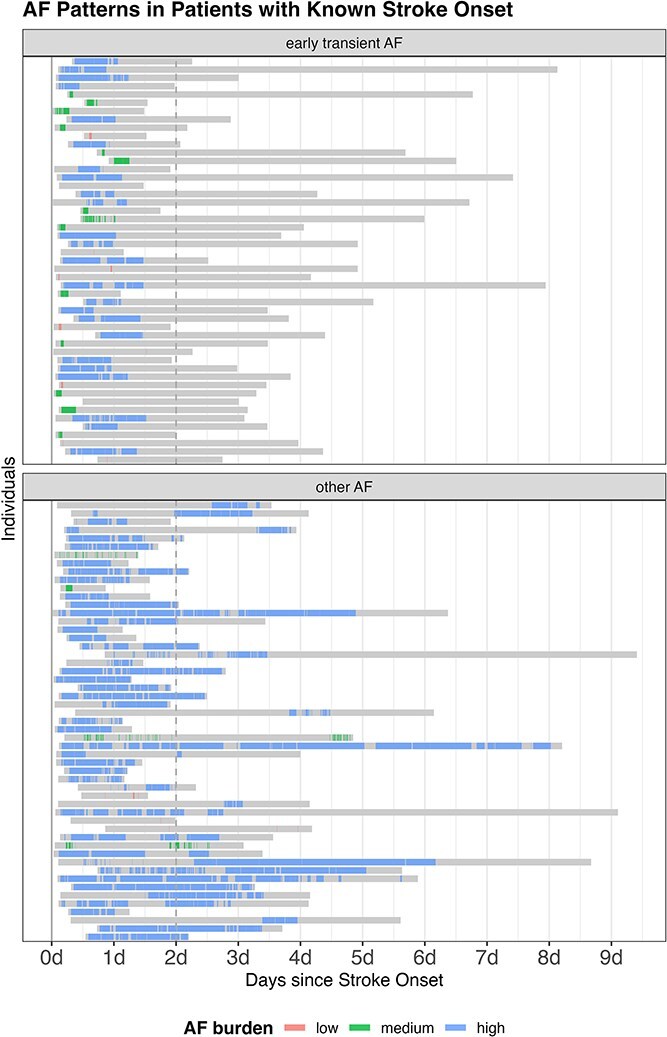
AF patterns in patients with known stroke onset (*n* = 102). Forty-nine patients had all AF episodes within the first 48 h post-stroke (“early transient AF”, top panel) and were more likely to have low (<1 h) or medium (1–6 h) AF burden. Each horizontal bar represents an individual: Grey shading indicates monitoring duration; coloured areas show AF episodes by burden group (see legend).

In patients admitted within 24 h of stroke (*n* = 102, median monitoring 68.9 h), almost half had AF exclusively in the first 48 h (“early transient AF”, *n* = 49), while others had no clear temporal pattern (“other AF”, *n* = 53; Figure 4). No significant differences in demographics or risk factors were observed (Tables S2 and S3), but early transient AF patients had numerically higher rates of insular stroke (30.6% vs. 20.8%, *P* = .27).

## Discussion

The primary aim of this study was to assess AF burden and episode dynamics after acute ischaemic stroke during continuous stroke unit ECG monitoring. We found marked heterogeneity in AF burden: nearly half of patients exhibited high burden (>6/24 h), while a substantial proportion showed no AF episodes despite a prior diagnosis. AF burden differed by insular stroke laterality, with lower burden in left-sided and higher burden in right-sided insular lesions. AF episodes in low/medium-burden patients clustered early after admission and in the late evening. In approximately half, AF terminated within 48 h, suggesting a transient post-stroke pattern.

These findings highlight considerable interindividual variability in AF burden and temporal dynamics in the acute post-stroke phase. The high prevalence of substantial AF burden may reflect advanced underlying disease, as AF burden increases with disease progression.^16^^,^^17^ However, similar cardiovascular comorbidity profiles across burden groups suggest that stroke-related factors may also contribute.

In contrast to prior studies based on long-term ECG monitoring,^5^^,^^8^^,^^18^ our analysis focused on the immediate post-stroke period, which may provide insight into early arrhythmia dynamics. While circadian heart rate alterations have been linked to stroke outcomes^14^ and AF occurrence,^19^ we did not observe striking differences in circadian patterns between burden groups. An exception is the fact that, in low AF burden patients, AF episodes were more likely to occur in the evening and early nighttime. This is in line with a small study showing that AFDAS was more likely in patients with nighttime stroke and could hint at a particular autonomic vulnerability in this time window that facilitates arrhythmia.^19^ The reasons for this finding in the present cohort, however, remain speculative.

The presented regression models suggest a lateralised association between insular stroke involvement and AF burden. Across both logistic and linear regression analyses, left-sided insular stroke was consistently associated with lower AF burden, whereas right-sided insular stroke showed a positive association that became more pronounced when modelled separately as a dichotomised variable (ie, right insular stroke versus left insular or non-insular stroke). Importantly, effect directions were consistent across model specifications, suggesting that differing significance levels reflect reference-group composition and statistical power rather than contradictory effects. These findings support the concept of hemispheric asymmetry in autonomic regulation after stroke, consistent with prior studies linking insular lesions to cardiac dysfunction, arrhythmia risk and specifically right insular stroke to worse cardiovascular prognosis.^9^^,^^10^^,^^20–22^ Interestingly, this right versus left laterality was not present in patients without insular lesions, highlighting the importance of the insular cortex regarding AF burden. Growing evidence suggests that the right and left insular cortex and their subregions fulfil different, but complementary functions, with the right dorsal anterior insular cortex most consistently associated with the parasympathetic nervous system, while the right ventral anterior and left posterior insular cortex are associated with the sympathetic nervous system.^23^ Hence, insular cortex lesions often follow a differential pattern depending on the affected side, with, for example, right (but not left) insular stroke resulting in decreased HRV and increased risk of sudden death.^9^ Importantly, right-sided insular stroke was associated with AFDAS in a Korean stroke population,^12^ strengthening the link between AF and insular stroke, even though other studies associated AFDAS more closely with left-sided insular stroke.^11^ A larger, adequately powered clinical trial comparing AF burden in insular vs. non-insular stroke patients could serve to confirm (or disprove) these findings.

In approximately half of patients, AF was confined to the first 48 h after stroke and did not recur during the remaining monitoring period (median 25 h), representing an early, and possibly transient AF pattern. Early transient AF was more common in patients with low or medium AF burden. Because pre-monitoring episodes were not captured, these patterns may be incomplete. However, the close temporal relationship between stroke onset and AF termination suggests a transient, stroke-related modulation of AF expression, potentially mediated by neurogenic mechanisms. This interpretation is supported by prior device-based studies showing similar temporal dynamics after stroke.^24^

Patients with early transient AF had numerically larger infarcts and more frequent insular involvement, although these differences were not statistically significant. While larger infarct size is associated with AFDAS,^25^ AFDAS prevalence was not higher in this subgroup, likely reflecting the inclusion of patients with known AF. These findings suggest stroke-related triggers may transiently modulate AF in pre-existing and newly detected cases alike—a hypothesis requiring prospective validation.

This study’s strengths include continuous ECG monitoring for precise AF burden and episode timing, and detailed imaging allowing adjustment for key confounders. Limitations include the single-centre retrospective design with potential for misclassification bias, unmeasured confounders and missing data, restriction to MRI-confirmed strokes, missing cardiac parameters (eg, echocardiography), occasional ECG data gaps, small subgroups for insular stroke analyses, lack of follow-up beyond hospitalisation and inability to distinguish isolated insular strokes from larger strokes extending into the insular cortex. It should also be noted that the burden classification applied in our analysis was originally proposed for prolonged cardiac monitoring. These factors may affect generalizability and interpretation of associations.

In this stroke cohort with real-world continuous stroke unit monitoring, AF burden and temporal episode patterns varied substantially in the acute post-stroke phase. Both burden and episode dynamics were associated with stroke characteristics, particularly insular involvement. Notably, AF burden differed by insular laterality, with lower burden in left-sided and higher burden in right-sided insular strokes. These findings may inform individualised monitoring strategies and support further investigation of stroke-related modulation of AF in prospective studies.

## Supplementary Material

rev_ecg_supplement_aakag079

## Data Availability

Data are available from the authors upon reasonable request and with permission of the local ethics committee.
