# Supplementary material for: Atrial fibrillation burden during in-patient cardiac monitoring after acute ischaemic stroke
Source: Eur Stroke J. 2026 Jul 21;11(7):aakag079. doi: 10.1093/esj/aakag079 (PMC13387441; doi:10.1093/esj/aakag079)
Supplement: rev_ecg_supplement_aakag079 [file rev_ecg_supplement_aakag079.docx]

Supplement:

Atrial fibrillation during in-patient cardiac monitoring after acute ischemic stroke

# Supplementary Methods:

## Atrial Fibrillation and Heart Rate Analysis

In AF patients, heart rate (HR) was calculated separately for AF episodes and for the periods between AF episodes. An AF episode was defined as a period spent in AF of at least 30 seconds in length and with at least 30 seconds interval to the next AF period to avoid overestimating the number of episodes. If the intervals between AF episodes were shorter, we subsumed them into one episode. If two alerts for AF episodes overlapped at the start or the end of each episode, we merged them into a single episode. Episodes recorded outside of the monitoring period were considered spurious data. For HR calculation, we used only continuous 5-minute segments, and only hours with at least three complete 5-minute segments were analyzed. Daytime was defined as 6 am to 10 pm, nighttime was defined as 10 pm to 6 am. The proportion of AF denotes the percentage of AF in relation to the total monitoring time. AF episodes were defined as bradycardic (mean HR < 60/s), normocardic (60-100/s) and tachycardic (> 100/s).

To categorize AF status, we differentiated patients with AF detected after stroke (AFDAS) and known AF. AFDAS was defined as AF newly diagnosed in in-patient cardiac monitoring. This included stroke unit monitoring and Holter monitoring. KAF was defined as any AF known to the patient and/or the physician before the index stroke *or* newly diagnosed AF detected on 12-lead admission ECG. Atrial flutter was categorized as either AFDAS or KAF, respectively. The categorization of AF status, along with other variables (NIHSS and modified Rankin Scale (mRS), intravenous thrombolysis, endovascular treatment, cancer status and smoking status, and prior use of oral anticoagulation was done manually by screening patient discharge letters. Cardiovascular risk factors (arterial hypertension, diabetes mellitus, coronary artery disease, dyslipidemia and history of myocardial infarction) were based on ICD-10 codes in patient charts which include known and newly diagnosed conditions.

For AF pattern analysis (early transient vs. other AF), only patients with known stroke onset admitted within 24 hours were included. Early transient AF was defined as: (1) episodes exclusively within 48 hours post-stroke, and (2) ≥24 h AF-free monitoring after the last episode (or ≥12 h for patients with AF burden <6 h). Patients not meeting both criteria were classified as “other AF.” Patients without recorded AF episodes or unclear stroke onset were excluded.

## Laboratory variables.

Recorded laboratory parameters included high-sensitivity cardiac Troponin T (hs-cTnT, 5^th^ generation Roche ElecSys Assay, 99^th^ percentile upper reference limit (URL) 14 ng/L), low-density lipoprotein (LDL) cholesterol and glomerular filtration rate (GFR) according to CKD-EPI formula. If multiple measurements were available, we chose the earliest recorded value after index stroke. To determine dynamic changes using serial hs-cTnT measurements, we ensured that the interval between the first and second measurement did not exceed 24 h. Acute myocardial injury was defined as dynamic hs-cTnT change ≥ 20 % increase or decrease between measurements with at least one value above the upper reference limit (URL).

## MRI variables.

MRI parameters like lesion pattern (single/scattered/multiple territories) and lesion type (embolic/lacunar) were assessed by a clinician with extensive stroke imaging experience (MGK). Single‐lesion pattern was defined as a singular lesion. Scattered‐lesion pattern was defined as nonsingular DWI lesion in either right anterior, left anterior, or posterior circulation (1). Stroke lesion volume was determined as described before (2).

# Supplementary Results:

## Regression modeling outputs.

All models used generalized linear models (binomial for logistic, Gaussian for linear). Below, the full model outputs are shown. log1p(nihss_a) is the log-transformed NIHSS on admission. rekaTRUE denominates obtained EVT. log1p(infarct_size) is the log-transformed stroke lesion volume. Insula_hemisphereLeft and insula_hemisphereRight are the side of insular stroke affection. SexM is male sex. Right_mca_affTRUE means stroke in the vascular territory of the middle cerebral artery (MCA), left_mca_affTRUE means left-sided stroke in MCA territory. Significant associations are highlighted in bold.

#### Univariate models: High AF Burden as dependent variable (logistic regression, only significant variables shown)

| Variable | OR | 95% CI Lower | 95% CI Upper | p-value |
| --- | --- | --- | --- | --- |
| **rekaTRUE** | **2.073953** | **1.306564** | **3.290620** | **0.001926782** |
| **insula_hemisphereRight** | **2.473333** | **1.365997** | **4.512220** | **0.002845276** |
| **log1p_nihss** | **1.302084** | **1.023494** | **1.664788** | **0.033085584** |

#### Multivariable logistic regression Model 1: High AF Burden as dependent variable (logistic regression)

| Variable | aOR | 95% CI Lower | 95% CI Upper | p-value |
| --- | --- | --- | --- | --- |
| (Intercept) | 0.7491142 | 0.07776551 | 6.9262536 | 0.80019533 |
| log1p(nihss_a) | 1.0758605 | 0.77657573 | 1.4924119 | 0.66011187 |
| **rekaTRUE** | **1.8130652** | **1.01125185** | **3.2656163** | **0.04613782** |
| log1p(infarct_size) | 1.0254802 | 0.85421338 | 1.2279805 | 0.78525954 |
| **insula_hemisphereLeft** | **0.3760674** | **0.15161690** | **0.8377715** | **0.02335482** |
| insula_hemisphereRight | 1.7565058 | 0.90366543 | 3.4167883 | 0.09543910 |
| age | 0.9932087 | 0.96660173 | 1.0209075 | 0.62397450 |
| sexM | 0.6977585 | 0.44676576 | 1.0845522 | 0.11115162 |

#### Right Insula Model 1: High AF Burden as dependent variable (right-sided insular stroke modeled against all other strokes incl. left-sided insular stroke)

| Variable | OR | 95% CI Lower | 95% CI Upper | p-value |
| --- | --- | --- | --- | --- |
| (Intercept) | 0.2586759 | 0.00490291 | 10.195092 | 0.481327772 |
| log1p(nihss_a) | 1.1876194 | 0.54899258 | 2.570803 | 0.658184711 |
| rekaTRUE | 1.4872909 | 0.49093764 | 4.564392 | 0.481139689 |
| log1p(infarct_size) | 1.0676661 | 0.73143144 | 1.565573 | 0.733719007 |
| **insular_aff_rightTRUE** | **5.4979565** | **2.01566056** | **16.421394** | **0.001319122** |
| age | 0.9933173 | 0.94844102 | 1.041898 | 0.777080134 |
| sexM | 0.4756770 | 0.16097712 | 1.307211 | 0.160492031 |
| right_mca_affTRUE | 0.5486242 | 0.13106075 | 2.124823 | 0.391157946 |

#### Left Insula Model 1: High AF Burden as dependent variable (left-sided insular stroke modeled against all other strokes incl. right-sided insular stroke)

| Variable | OR | 95% CI Lower | 95% CI Upper | p-value |
| --- | --- | --- | --- | --- |
| (Intercept) | 1.0587563 | 0.01914572 | 52.693555 | 0.977145908 |
| log1p(nihss_a) | 1.1393452 | 0.52051928 | 2.490028 | 0.740660415 |
| rekaTRUE | 1.8479561 | 0.60890203 | 5.852337 | 0.283210999 |
| log1p(infarct_size) | 1.0717547 | 0.73394418 | 1.572403 | 0.719188840 |
| **insular_aff_leftTRUE** | **0.1935275** | **0.06596930** | **0.511037** | **0.001486102** |
| age | 0.9938353 | 0.94807768 | 1.043360 | 0.797829681 |
| sexM | 0.5330893 | 0.18310649 | 1.468046 | 0.232285992 |
| left_mca_affTRUE | 2.2865564 | 0.35398143 | 13.576306 | 0.362346202 |

#### Univariate Models: log -transformed AF burden as dependent variable (linear regression, only significant variables shown)

| Variable | β | 95% CI Lower | 95% CI Upper | p-value |
| --- | --- | --- | --- | --- |
| **age** | **0.0799** | **0.0283** | **0.132** | **0.00249** |
| **sexM** | **-1.01** | **-1.87** | **-0.145** | **0.0222** |
| **lyseTRUE** | **1.16** | **0.0445** | **2.28** | **0.0416** |
| **rekaTRUE** | **1.09** | **0.0978** | **2.09** | **0.0314** |
| **insula_hemisphereRight** | **1.32** | **0.0162** | **2.62** | **0.0472** |

#### Continuous-scale regression results

In univariate linear regression, age, female sex, IVT, EVT and right insular stroke were positively associated with higher log AF burden (all p<0.05). Fully adjusted multivariate models confirmed the association of higher log AF burden with age (β 0.07, 95%CI 0.02–0.12, p<0.05) and IVT (β 1.14, 95%CI 0.02–2.25, p<0.05). Insular stroke as a categorical variable was non-significant in the primary multivariable model (Model 2, see below). When modeled as a dichotomized variable (right insula vs. all other strokes; left insula vs. all other strokes), we observed a positive association with higher AF burden for right-sided insular stroke (β 2.34, 95%CI 0.44–4.24, p<0.05) and a negative association for left-sided insular stroke (β −1.97, 95%CI −3.8–(−0.13), p<0.05).

#### Multivariable linear regression Model 2: Log-transformed AF Burden as dependent variable (linear regression)

| Variable | β | 95% CI Lower | 95% CI Upper | p-value |
| --- | --- | --- | --- | --- |
| **lyseTRUE** | **1.137** | **0.023** | **2.252** | **0.045** |
| rekaTRUE | 0.583 | -0.480 | 1.646 | 0.282 |
| log1p(infarct_size) | 0.105 | -0.241 | 0.450 | 0.552 |
| insula_hemisphereLeft | -0.701 | -2.162 | 0.761 | 0.347 |
| insula_hemisphereRight | 0.919 | -0.477 | 2.315 | 0.196 |
| **age** | **0.069** | **0.016** | **0.121** | **0.01** |
| sexM | -0.767 | -1.644 | 0.110 | 0.086 |
| log1p(mon_len) | 0.217 | -0.157 | 0.592 | 0.255 |

#### Right Insula Model 2: Log-transformed AF Burden as dependent variable (right-sided insular stroke modeled against all other strokes incl. left-insular stroke, linear regression)

| Variable | β | 95% CI Lower | 95% CI Upper | p-value |
| --- | --- | --- | --- | --- |
| lyseTRUE | 1.145 | -1.059 | 3.348 | 0.305 |
| rekaTRUE | -0.690 | -2.596 | 1.215 | 0.473 |
| log1p(infarct_size) | 0.266 | -0.400 | 0.933 | 0.429 |
| **insular_aff_rightTRUE** | **2.338** | **0.437** | **4.239** | **0.017** |
| age | 0.025 | -0.059 | 0.110 | 0.549 |
| sexM | -1.335 | -3.257 | 0.587 | 0.171 |
| log1p(mon_len) | 0.008 | -0.910 | 0.926 | 0.987 |
| right_mca_affTRUE | -2.534 | -5.266 | 0.198 | 0.069 |

#### Left Insula Model 2: Log-transformed AF Burden as dependent variable (left-sided insular stroke modeled against all other strokes incl. right-sided insular stroke, linear regression)

| Variable | β | 95% CI Lower | 95% CI Upper | p-value |
| --- | --- | --- | --- | --- |
| lyseTRUE | 1.058 | -1.163 | 3.279 | 0.346 |
| rekaTRUE | -0.089 | -2.016 | 1.839 | 0.927 |
| log1p(infarct_size) | 0.264 | -0.408 | 0.936 | 0.436 |
| **insular_aff_leftTRUE** | **-1.968** | **-3.808** | **-0.129** | **0.036** |
| age | 0.025 | -0.060 | 0.110 | 0.561 |
| sexM | -1.070 | -3.022 | 0.882 | 0.279 |
| log1p(mon_len) | 0.044 | -0.885 | 0.972 | 0.925 |
| left_mca_affTRUE | 2.315 | -1.065 | 5.696 | 0.177 |

## Heart rate and episode characteristics.

HR during AF was highest in the medium-burden group (92.5 vs. 83.1 and 82.1 bpm, p<0.05). Mean hourly HR showed peaks at 7 am and 3–4 pm, with no significant differences between groups; median sinus rhythm HR was numerically lowest in low-burden patients. High-burden patients had fewer tachycardic episodes (20.8%) and 63.5% normocardic AF episodes, while medium-burden patients had more tachycardic episodes (34.3%, p<0.05). Bradycardic episodes were rare in all groups (Fig. 2C).

## Table S1:

Table S1: Baseline characteristics of AF patients stratified by AF burden (high vs. low AF burden)

| **Variable** | **All** | | | **AF burden** | | | | | | | | **p value** | |
| --- | --- | --- | --- | --- | --- | --- | --- | --- | --- | --- | --- | --- | --- |
|  |  |  |  | **< 6/24h** | | | | **>= 6/24h** | | | |  |  |
| N | 392 (100) | | | 199 (50.8) | | | | 193 (49.2) | | | |  | |
| Age (y) | 82 (77-86) | | | 81 (77-86) | | | | 82 (76-86) | | | | 0.71 | |
| Female | 210 (53.6) | | | 102 (51.3) | | | | 108 (56) | | | | 0.41 | |
| Length of monitoring (h) | 68.3 (41.7-98.8) | | | 64.6 (40.1-99.9) | | | | 70.5 (44.1-97.3) | | | | 0.45 | |
| NIHSS on admission | 4 (2-9) | | | 3 (1-8) | | | | 5 (2-11) | | | | < 0.01 | |
| IVT | 72 (18.4) | | | 34 (17.1) | | | | 38 (19.7) | | | | 0.59 | |
| EVT | 98 (25) | | | 37 (18.6) | | | | 61 (31.6) | | | | < 0.01 | |
| **Cardiovascular risk factors** | |  | | |  | | | |  | | | |  |
| Arterial hypertension | 313 (79.8) | | | 158 (79.4) | | | | 155 (80.3) | | | | 0.92 | |
| Diabetes mellitus | 97 (24.7) | | | 47 (23.6) | | | | 50 (25.9) | | | | 0.68 | |
| Dyslipidemia | 270 (68.9) | | | 141 (70.9) | | | | 129 (66.8) | | | | 0.45 | |
| Coronary artery disease | 74 (18.9) | | | 46 (23.1) | | | | 28 (14.5) | | | | < 0.05 | |
| History of myocardial infarction | 31 (7.9) | | | 17 (8.5) | | | | 14 (7.3) | | | | 0.78 | |
| Ipsilateral ICA stenosis >50% | 21 (5.4) | | | 15 (7.5) | | | | 6 (3.1) | | | | 0.07 | |
| CHA2DS2-VA score | 3 (3-4) | | | 3 (3-4) | | | | 3 (2-4) | | | | 0.28 | |
| Known AF | 312 (79.6) | | | 157 (78.9) | | | | 155 (80.3) | | | | 0.82 | |
| AFDAS | 80 (20.4) | | | 42 (21.1) | | | | 38 (19.7) | | | | 0.82 | |
| Active intake of OAC | 190 (48.5) | | | 94 (47.2) | | | | 96 (49.7) | | | | 0.69 | |
| Admission hs-cTnT (ng/L) | 24 (15-39.8) | | | 24 (15-42) | | | | 23 (15-35) | | | | 0.43 | |
| Acute myocardial injury | 88 (22.4) | | | 50 (25.1) | | | | 38 (19.7) | | | | 0.24 | |
| **Laboratory values** | | |  | | |  | | | |  | | |  |
| CRP (mg/L) | 3.6 (1.6-11.5) | | | 3.6 (1.4-10.4) | | | | 3.7 (1.7-14.1) | | | | 0.31 | |
| Leukocytes (10^9/L) | 8.7 (7-10.9) | | | 8.7 (6.9-10.9) | | | | 8.7 (7-10.9) | | | | 0.78 | |
| GFR (mL/min/1.73m²) | 59 (45-76) | | | 60 (46-77) | | | | 58.5 (43.8-74.2) | | | | 0.25 | |
| **HR pattern** | | |  | | |  | | | |  | | |  |
| SVES in 24 h | 678.1 (182-1556) | | | 410 (87.7-1423.5) | | | | 910.3 (337-1620.8) | | | | < 0.001 | |
| HR overall | 75.7 (67.4-87.6) | | | 73.8 (66.4-84.3) | | | | 78.2 (69.6-89.9) | | | | < 0.01 | |
| **Stroke characteristics on MRI** | | |  | | | |  | | | |  | |  |
| Right anterior circulation stroke | 98 (25) | | | 45 (22.6) | | | | 53 (27.5) | | | | 0.34 | |
| Left anterior circulation stroke | 91 (23.2) | | | 48 (24.1) | | | | 43 (22.3) | | | | 0.73 | |
| Posterior circulation stroke | 70 (17.9) | | | 32 (16.1) | | | | 38 (19.7) | | | | 0.44 | |
| Stroke in multiple vascular territories | 132 (33.7) | | | 73 (36.7) | | | | 59 (30.6) | | | | 0.23 | |
| Hemorrhagic transformation | 92 (23.5) | | | 42 (21.1) | | | | 50 (25.9) | | | | 0.33 | |
| Stroke lesion volume (mL) | 2.4 (0.4-12.2) | | | 1.7 (0.3-8.7) | | | | 3.2 (0.4-16.8) | | | | < 0.05 | |
| Highest quartile of lesion volume | 134 (34.2) | | | 59 (29.6) | | | | 75 (38.9) | | | | 0.05 | |
| Right insular stroke | 52 (13.3) | | | 18 (9) | | | | 34 (17.6) | | | | < 0.05 | |
| Left insular stroke | 45 (11.5) | | | 27 (13.6) | | | | 18 (9.3) | | | | < 0.05 | |
| *Summary statistics are displayed as median (IQR) or n (%).* | | | | | | | | | | | | | |

Table S1: Baseline characteristics stratified by AF burden under or over 6/24 h. NIHSS: National Institutes of Health Stroke Scale. IVT: Intravenous thrombolysis. EVT: Endovascular treatment. OAC: Oral anticoagulants. AF: Atrial fibrillation. AFDAS: Atrial Fibrillation Detected After Stroke. hs-cTnT: High-sensitivity cardiac troponin T. CRP: C-reactive protein. GFR: Glomerular filtration rate. SVES: Supraventricular extrasystoles. HR: Heart rate. MRI: magnetic resonance imaging. ICA: internal carotid artery.

## Table S2:

Table S2: Characteristics of patients with early transient vs. other AF

| **Variable** | | | **AF pattern** | |  |
| --- | --- | --- | --- | --- | --- |
|  |  |  | **early transient**  **N = 49** | **Other**  **N = 53** | **P value** |
| Age (y) | | | 82 (76-86) | 81 (74-85) | 0.65 |
| Female | | | 25 (51) | 31 (58.5) | 0.55 |
| NIHSS on admission | | | 4 (2-9) | 3 (1-6) | 0.27 |
| IVT | | | 15 (30.6) | 8 (15.1) | 0.1 |
| EVT | | | 15 (30.6) | 12 (22.6) | 0.38 |
| **Cardiovascular risk factors** |  |  |  |  |  |
| Arterial hypertension | | | 42 (85.7) | 43 (81.1) | 0.6 |
| Diabetes mellitus | | | 13 (26.5) | 12 (22.6) | 0.82 |
| Dyslipidemia | | | 37 (75.5) | 35 (66) | 0.38 |
| Coronary artery disease | | | 7 (14.3) | 12 (22.6) | 0.32 |
| CHA2DS2-VASc score | | | 4 (3-4) | 3 (3-4) | 0.73 |
| Ipsilateral ICA stenosis > 50 % | | | 1 (2) | 3 (5.7) | 0.62 |
| History of stroke | | | 2 (4.1) | 3 (5.7) | 1 |
| History of myocardial infarction | | | 4 (8.2) | 7 (13.2) | 0.53 |
| **Stroke characteristics on MRI** |  |  |  |  |  |
| Stroke lesion volume (mL) | | | 1.7 (0.2-9.2) | 0.8 (0.2-5.5) | 0.25 |
| Top 25% lesion volume | | | 18 (36.7) | 17 (32.1) | 0.63 |
| Embolic lesion | | | 49 (100) | 49 (92.5) | 0.12 |
| Lacunar lesion | | | 0 (0) | 4 (7.5) | 0.12 |
| Insular stroke | | | 15 (30.6) | 11 (20.8) | 0.27 |
| Right-sided infarct | | | 8 (16.3) | 12 (22.6) | 0.46 |
| Left-sided infarct | | | 14 (28.6) | 14 (26.4) | 0.83 |
| Posterior infarct | | | 9 (18.4) | 12 (22.6) | 0.63 |
| Multiple infarcts | | | 18 (36.7) | 15 (28.3) | 0.4 |
| Hemorrhagic transformation | | | 15 (30.6) | 9 (17) | 0.16 |
| **Laboratory variables** | |  |  |  |  |
| Admission hs-cTnT (ng/L) | | | 23 (16-32) | 21 (15-36) | 0.76 |
| GFR (mL/min/1.73m²) | | | 55 (41-65) | 58 (40-71) | 0.56 |
| CRP (mg/L) | | | 3.2 (1.6-8.3) | 2.4 (1.3-9.4) | 0.63 |
| LDL (mg/dL) | | | 97 (76-124) | 81 (61.5-116.5) | 0.06 |
| Leukocytes (10^9/L) | | | 8.5 (7.4-10.4) | 8.2 (6.7-9.3) | 0.13 |
| *Summary statistics are displayed as median (IQR) or n (%).* | | | | | |

**Table S2.** NIHSS: National Institutes of Health Stroke Scale. IVT: Intravenous thrombolysis. EVT: Endovascular treatment. OAC: Oral anticoagulants. AF: Atrial fibrillation. AFDAS: Atrial Fibrillation Detected After Stroke. hs-cTnT: High-sensitivity cardiac troponin T. CRP: C-reactive protein. LDL: Low-density lipoprotein. GFR: Glomerular filtration rate. ICA: internal carotid artery.

## Table S3:

Table S3: Summary of Cardiac Monitoring Parameters and AF Characteristics Stratified by AF Pattern

| **Variable** | **AF pattern** | | **p: overall** |
| --- | --- | --- | --- |
|  | **early transient** | **other** |  |
| Patient count | 49 (48.04) | 53 (51.96) |  |
| Time spent in AF over 24h (hours) | 2.4 (0.6-4.6) | 11.9 (4.9-18.2) | < 0.001 |
| AF burden: low | 10 (20.4) | 3 (5.7) | < 0.05 |
| AF burden: medium | 13 (26.5) | 4 (7.5) | < 0.05 |
| AF burden: high | 26 (53.1) | 46 (86.8) | < 0.001 |
| Number of episodes | 4 (2-8) | 17 (10-26) | < 0.001 |
| Median length of AF episode (minutes) | 83 (43.6-139.3) | 100.8 (55-140) | 0.6 |
| Shortest AF episode (minutes) | 5.8 (1.5-23) | 5.7 (2.8-8.2) | 0.79 |
| Longest AF episode (minutes) | 236.8 (90.9-425) | 364.3 (182.7-581.2) | < 0.05 |
| AF burden per 24 h in % | 9.9 (2.7-19) | 49.7 (20.3-75.8) | < 0.001 |
| Overall HR during AF episodes | 78.1 (67.7-94) | 76.4 (67.4-85.7) | 0.64 |
| HR during daytime AF episodes | 80.2 (68.2-99.5) | 79.7 (71.2-89.3) | 0.94 |
| HR during nighttime AF episodes | 78.1 (68.4-99) | 75.6 (69.2-86.8) | 0.47 |
| Overall HR during SR | 74.6 (66.6-83.1) | 73 (66.2-79.8) | 0.71 |
| HR during daytime SR | 76.3 (68.4-84.2) | 74.6 (68.2-84.4) | 0.53 |
| HR during nighttime SR | 74.9 (67.2-83.2) | 73.4 (66.2-81.5) | 0.63 |
| % of AF episodes with normal HR | 60.8 (40.7-71.2) | 67.4 (54.3-76.3) | < 0.05 |
| % of AF episodes with bradycardia | 12 (2.5-26.3) | 13.2 (4.9-26.3) | 0.59 |
| % of AF episodes with tachycardia | 14.5 (1.6-38.3) | 7.2 (1.1-25.9) | 0.37 |
| Known AF | 42 (85.7) | 43 (81.1) | 0.6 |
| AFDAS | 7 (14.3) | 10 (18.9) | 0.6 |
| Time to first AF episode after stroke onset (hours) | 7.2 (3.4-13.4) | 7.4 (4.3-18.8) | 0.16 |
| Time to cardiac monitoring after stroke onset (hours) | 4.2 (3-10.4) | 5.4 (3.8-8.5) | 0.29 |
| *Summary statistics are displayed as median (IQR) or n (%).* | | | |

**Table S3.** AF: Atrial fibrillation. HR: Heart rate. SR: Sinus rhythm. AFDAS: AF detected after stroke.

# References

1. Braemswig TB, Usnich T, Albach FN, et al. Early new diffusion-weighted imaging lesions appear more often in stroke patients with a multiple territory lesion pattern. *Stroke*. Aug 2013;44(8):2200–4. doi:10.1161/STROKEAHA.111.000810
2. Klammer MG, Reimann L, Richter O, et al. Association Between Stroke Lesion Size and Atrial Fibrillation Detected After Stroke: An Observational Cohort Study. *J Am Heart Assoc*. Oct 2024;13(19):e035285. doi:10.1161/JAHA.124.035285
